# Epidemiological investigations of human rabies in China

**DOI:** 10.1186/1471-2334-9-210

**Published:** 2009-12-21

**Authors:** Miao Song, Qing Tang, Ding-Ming Wang, Zhao-Jun Mo, Shou-Heng Guo, Hao Li, Xiao-Yan Tao, Charles E Rupprecht, Zi-Jian Feng, Guo-Dong Liang

**Affiliations:** 1 State Key Laboratory for Molecular Virology and Genetic Engineering, National Institute for Viral Disease Control and Prevention, Chinese Center for Disease Control and Prevention, Beijing 100052, PR China; 2 Liupanshui Vocational and Technical College, Liupanshui 553001, PR China; 3 Guizhou Center for Disease Control and Prevention, Guiyang 550004, PR China; 4 Guangxi Center for Disease Control and Prevention, Nanning 530028, PR China; 5 Hunan Center for Disease Control and Prevention, Changsha 410005, PR China; 6 National Center for Zoonotic, Vector-Borne and Enteric Diseases, Centers for Disease Control and Prevention, Atlanta, GA. 30333, USA; 7 Office for Disease Control and Emergency Response, Chinese Center for Disease Control and Prevention, Beijing 100050, PR China; 8 State Key Laboratory for Infectious Disease Prevention and Control, National Institute for Viral Disease Control and Prevention, Chinese Center for Disease Control and Prevention, Beijing 100052, PR China

## Abstract

**Background:**

The epidemic of rabies showed a rising trend in China in recent years. To identify the potential factors involved in the emergence, we investigated and analyzed the status and characteristics of human rabies between 1996 and 2008. Moreover, the status of rabies infection and vaccination in dogs, and prophylaxis of humans after rabies exposure were analyzed.

**Methods:**

Human rabies data in China between 1996 and 2008 collected from the annual reports of Chinese Center for Disease Control and Prevention (China CDC) were analyzed. To investigate the status of dogs and postexposure prophylaxis (PEP) of humans, brain specimens of domestic dogs were collected and detected, and the demographic details, exposure status and PEP of rabies patients were obtained in 2005 and 2006 in Guangxi, Hunan and Guizhou provinces.

**Results:**

The results showed 19,806 human rabies cases were reported in China from 1996 to 2008, with an average of 1,524 cases each year, and the incidence almost was rising rapidly, with the peak in 2007 (3,300 cases). It was notable that nearly 50% of the total rabies cases nationwide were reported in Guangxi, Hunan and Guizhou provinces. In these three provinces, the rabies infection rate in dogs was 2.3%, and 60% investigated cities had a dog vaccination rate of below 70%; among the 315 recorded human cases, 66.3% did not receive any PEP at all, 27.6% received inadequate PEP, and only 6.0% received a full regime of PEP.

**Conclusions:**

In recent years, rabies is reemerging and becoming a major public-health problem in China. Our analysis showed that unsuccessful control of dog rabies and inadequate PEP of patients were the main factors leading to the high incidence of human rabies in China, then there are following suggestions: (1) Strict control of free-ranging dogs and mandatory rabies vaccination should be enforced. (2)Establishing national animal rabies surveillance network is imperative. (3) PEP should be decided to initiate or withhold according to postmortem diagnosis of the biting animal. (4) The cost of PEP should be decreased or free, especially in rural areas. (5)Education of the public and health care staff should be enhanced.

## Background

Rabies is widely distributed across the globe. More than 55,000 people die of rabies each year [1]. About 95% of human deaths occur in Asia and Africa. India is the most severely affected, and China is the next. In the 1980s, rabies was a serious problem in China, with thousands of human cases annually, except for a brief decline in the middle of the 1990s [2]. The historically lowest incidence of human rabies (159 cases) was recorded in 1996, followed by a rapidly rising trend of cases [3], which is paid serious attention in China. Han Si et al. conducted a comprehensive analysis of the rabies situation in China from 1990 to 2007 and analysed the reasons for the post-exposure treatment failures based on human rabies cases records in Guangdong province [4]. To identify the potential factors involved in recent rabies emergence and provide reliable date as the basis for the development of an appropriate prevention plan, we investigated and analyzed the status and characteristics of human rabies between 1996 and 2008. Moreover, we focused upon the three most seriously affected provinces (Guangxi, Hunan and Guizhou), in which the status of rabies infection and vaccination in dogs, and prophylaxis of humans after rabies exposure were analysed.

## Methods

### Data resources

Human rabies data in China between 1996 and 2008, including those of Guangxi, Hunan and Guizhou provinces, were collected from the annual reports of Chinese Center for Disease Control and Prevention (China CDC). The data on vaccination rates of local dogs between 2005 and 2006 in the three provinces were gathered from the local Veterinary Departments. The vaccination coverage rates of dogs were estimated by the number of vaccination dogs/the number of all dogs. The demographic details of rabies patients (name, gender, age, occupation), exposure status (exposure site and degree) and postexposure prophylaxis (PEP) of the patients in 2005 and 2006 in Guangxi, Hunan and Guizhou provinces were obtained from the individual case reports of the three provincial CDC. (Registry data and veterinary data sets employed in this study were approved by National Institute for Viral Disease Control and Prevention, China CDC, and Office for Disease Control and Emergency Response, China CDC. Director: Feng Zijian, fengzj@chinacdc.cn)

PEP is recommended for postexposure rabies prophylaxis by World Health Organization (WHO) criteria [5]. In 2007, China CDC published Treatment Guidelines for Rabies Postexposure Prophylaxis of Humans based on the WHO criteria above. Appropriate wound care of immediate and thorough washing of the wound for at least 15 minutes using soap and water, and rabies vaccine administration are needed for Category II and III contacts. In addition, rabies immune globulin (RIG) is administered for Category III contacts. There are two types of RIG used in China, equine RIG (purified rabies equine immunoglobulin) is used more frequently than human RIG (rabies human immunoglobulin). The dose of equine RIG is 40 international unit (IU)/kg of body weight and the dose for human RIG is 20 IU/kg of body weight. Injection of cell culture rabies vaccines is on each of days 0, 3, 7, 14 and 28.

### Investigation of the status of rabies infection in dogs

**Figure 1:**
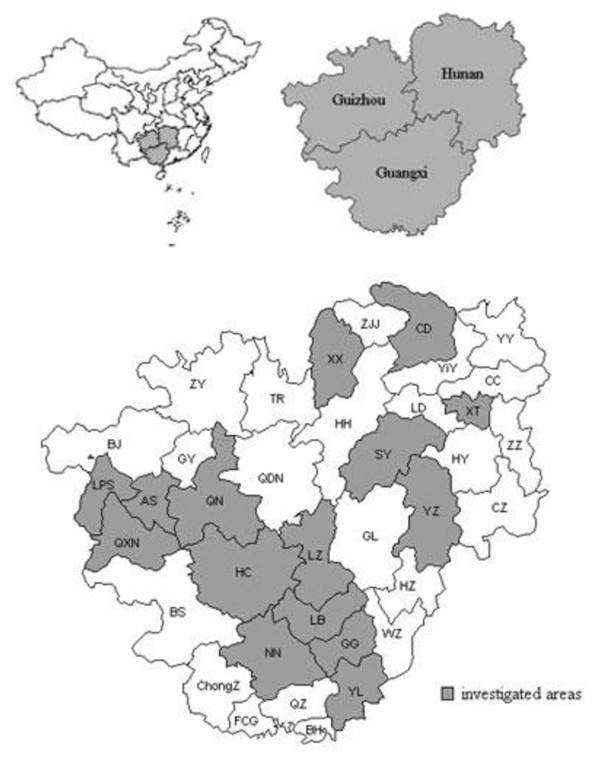
**Location of Guangxi, Hunan and Guizhou provinces in China and data on human cases and dogs was included for highlighted counties**.

Based on the epidemiological situation of rabies in 2004, 15 principal cities with a different epidemic status in the three provinces (Guangxi, Hunan and Guizhou) were selected (Figure 1). There are many restaurants selling dog meat here and there, for people in southern China are accustomed to eat dog meat to resist dampness and cold in winter, and almost all the dogs collected for meat consumption are local domestic dogs free-ranging and unvaccinated in rural areas which are majorly accounted for human rabies. So the brain specimens from apparently healthy dogs in different restaurants in the 15 principal cities can be regarded as from random domestic dogs. From October 2005 to July 2006, 2887 brain specimens were collected by the local CDC.

The direct fluorescent-antibody assay (DFA), the traditional global standard procedure for rabies diagnosis approved by WHO [6], was used to detect all the samples of dog brain tissue with fluorescence-labeled monoclonal antibody directed against the rabies virus N-protein (Rabies DFA Reagent; Chemicon Europe Ltd, Chandlers Ford, Southampton, UK) [7].

### Data analysis

Descriptive statistics were used to analyze the characteristics and the dynamics of rabies between 1996 and 2008 in China and the three selected provinces. For human rabies, once clinical signs appear, the disease is essentially 100% fatal. So the mortality is identical to the incidence rate [4], and incidence rate was calculated in this study. The status of rabies infection and vaccination in dogs, pattern of rabies exposures and the rates of PEP in the recent two years were analyzed separately. A chi-square test was applied to analyze the differences in the DFA positive rates of rabies among Hunan, Guangxi and Guizhou provinces. A linear correlation was applied to analyze the relationship among the DFA positive rate of rabies in dogs, the incidence of human rabies and the rate of vaccination in dogs. In addition, a logistic regression model was used to analyze the factors influenced PEP.

### Statistical software

Excel 2003, MapInfo 7.0 and SPSS 13.0 were used for statistical analysis.

## Results

### Demographic characteristics and dynamics of human rabies in China from 1996 to 2008

**Figure 2:**
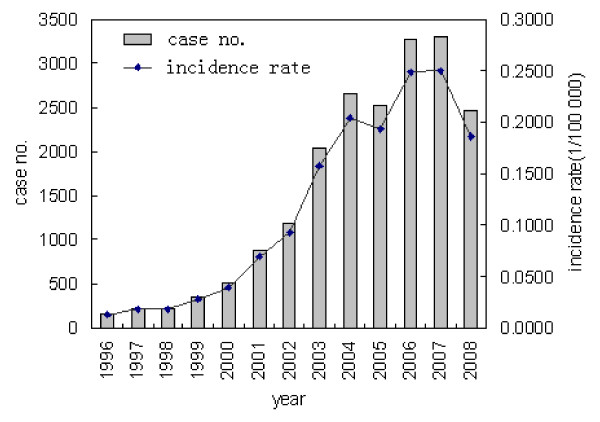
**Number of human cases and incidence rate between 1996 and 2008 in China**.

A total of 19,806 human rabies cases were reported in China from 1996 to 2008, with an average of 1,524 cases each year and an incidence rate of 0.1189 per 100,000. The fewest cases (159) occurred in 1996, after which the number of reported cases nearly increased each year until 2007, with 3,300 cases reported, 20.8 times as many as in 1996. In particular, the case number increased 75.6% in 2001 compared to 2000, and in 2003 an increase of 846 cases was reported compared to 2002, which had a 71.0% increase. In 2005, the number of cases decreased, with a reduction of 103 cases. However, an increase of 745 cases in 2006 was reported compared to 2005, an increase of 29.2%. The second reduction of rabies cases in the 13 years was reported in 2008(2478), even lower than the cases in 2004(Figure 2).

During these 13 years, 29 of the 31 provinces in China (except Tibet and Qinghai) reported human rabies cases. The ten provinces reported the most cases were, in decreasing order, Guangxi, Hunan, Guizhou, Guangdong, Jiangxi, Jiangsu, Hubei, Henan, Sichuan and Anhui. There were 17,210 human cases reported in these ten provinces, mainly located in the middle and southeast China, accounting for 86.9% of the national reporting. The geographical distribution was uneven. In 1996, there were only five provinces with more than 10 rabies cases, which increased to nine provinces in 2001 and to 17 provinces in 2006 and 2007. Initially and until now, most of the affected provinces were localized in the eastern, southern and central China, but the prevalence area began to expand into the western and northern China in recent years.

The demographic characteristics of all the human rabies cases in China from 1996 to 2008 were presented in Table 1. Male cases were more than twice as frequent as female cases. Nearly 1/4 of the cases were under 15 years old. In all the patients, farmers (rural agricultural workers) represented 63.4%, much more than the other occupations. Rabies cases occurred in all four seasons but with an autumnal peak.

**Table 1:** The demographic characteristics of all the human rabies cases in China (n = 19,806) and in the three affected provinces (Guangxi, Hunan and Guizhou, n = 9,130) from 1996 to 2008

Categories	Nation (%)	Three provinces (%)
Sex		
Male	68.9	69.6
Female	31.1	30.4

Age		
<15	25.0	27.1
15~	9.3	9.7
30~	18.8	18.3
45~	25.9	24.2
60~	8.3	17.2
75~	13.0	3.5

Occupation		
Peasants	63.4	63.9
Students	17.3	18.9
Unattended children	8.4	9.2
Others	10.9	8.0

Season		
Spring	19.5	20.4
Summer	29.5	29.4
Fall	31.7	30.9
Winter	19.3	19.4

### Demographic characteristics and dynamics of human rabies in three affected provinces from 1996 to 2008

**Figure 3:**
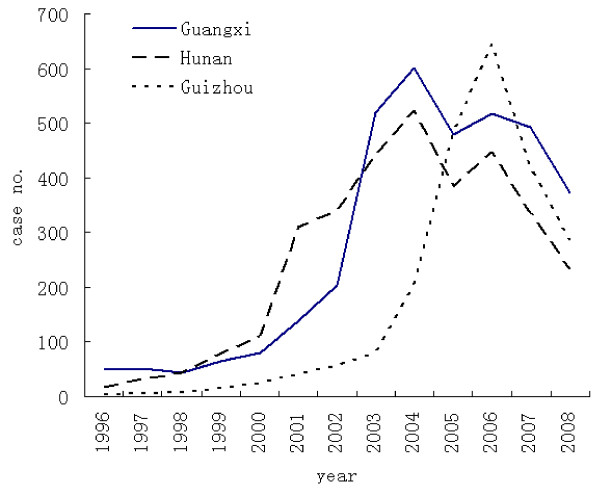
**Changes in the number of cases of human rabies between 1996 and 2008 in Guangxi, Hunan and Guizhou provinces**.

A total of 9,130 human rabies cases were reported from 1996 to 2008 in the highest prevalence provinces of Guangxi, Hunan and Guizhou, accounting for 46.1% of the total cases in China. Of these, 3,563 cases were in Guangxi, 3,294 in Hunan, and 2,273 in Guizhou. The incidence rate of human rabies was 0.4261/100,000 in Guangxi, 0.5363/100,000 in Hunan, and 0.4686/100,000 in Guizhou. Human rabies incidence rose over the past 13 years in the three provinces (Figure 3). The year with the highest increase of cases compared to the previous year was 2003 for Guangxi (154.4% increase compared to 2002), 2001 for Hunan (180.2% increase compared to 2000), and 2005 for Guizhou (134.5% increase compared to 2004).

Over the 13 years, the incidence increase and prevalence spread continued in the three provinces, with 15 cities reported human rabies cases in 1996, 24 cities in 2001, and all 37 cities after 2004. There were relatively more cases in the southwest of Guizhou, south of Hunan and southeast of Guangxi than in the other regions, and cases were mostly distributed in the rural areas. The demographic characteristics of human rabies cases at the provincial level were the same as the national level (Table 1).

### Status of rabies infection and vaccination in dogs

In 2005 and 2006, 2,887 dog brains were tested for rabies virus antigen by DFA in the three provinces (Figure 1). The infection rate of rabies is 2.3% of dogs tested. Of these, an infection rate is 1.9% in Guangxi, 2.6% in Hunan, and 2.6% in Guizhou. All 15 cities showed an infection rate of 0.0-4.6% (Table 2). The positive rate showed no significant difference among the three provinces (χ^2 ^= 1.508, *P *= 0.471).

**Table 2:** The infection rate and vaccination rate in dogs and incidence of human rabies in 15 investigated cities

Investigated cities	Infection rate of dogs (%)	Vaccination rate of dogs (%)	Incidence of human cases (1/100,000)
LB	1.9	85.2	2.67
GG	2.6	93.0	1.00
HC	0.5	26.5	0.78
YL	1.3	89.0	1.00
NN	3.2	73.2	1.12
LZ	1.9	10.0	1.57
YZ	1.2	19.6	0.63
SY	4.0	15.0	0.55
XT	2.6	33.0	0.72
CD	3.9	-	0.51
XX	2.0	-	0.30
QXN	3.0	-	4.39
QN	1.9	13.0	1.59
AS	4.6	-	2.84
LPS	0.0	-	0.46

The status of dog vaccination was collected in 10 of the 15 cities with dog rabies testing data (Table 2, Figure 1). Only four cities (40%) had a vaccination rate of over 70%. The highest rabies vaccination rate was in GG (93.0%), and the lowest rate was in LZ (10.0%).

Analysis of the correlation between the infection rate in dogs and the incidence of human rabies in these cities showed a non-significant relationship (*r *= 0.172, *P *= 0.540) (Table 2). There is also a non-significant relationship (*r *= 0.303, *P *= 0.394) between the incidence of human rabies and the vaccination rate of dogs, and between the infection rate in dogs and the vaccination rate of dogs (*r *= 0.549, *P *= 0.100).

### Rabies PEP

We collected detailed information on 711 of the human 2956 rabies cases from Guangxi, Hunan and Guizhou provinces in 2005 and 2006(24.1%). Of the 711 recorded cases, 91.0% were attacked by dogs, 6.5% by cats and the remainder by other animals. In these dogs, 2.81% had been vaccinated, and all the cats were unvaccinated. According to the category of contact with animals by WHO, 6.3% of the patients had category I contacts (i.e., touching or feeding of animals, or licks upon intact skin), 30.4% had category II contacts (i.e., nibbling of uncovered skin, or minor abrasions without bleeding), and 63.3% had category III contacts (transdermal bites or scratches, or mucous membrane contamination). In the 631 cases with exposure site recorded, 15.1% had exposures to the head, face or neck, 1.0% on the trunk, 48.2% on the upper limbs and 35.8% on the lower limbs.

Among the 711 recorded human cases, 315 cases had sufficient information of PEP, which were analyzed in detail. Of these, 209 cases (66.3%) did not receive medical services at all; 41 cases only received treatment of the wounds; 14 cases only received rabies vaccine; 32 cases with category III exposure only had treatment of the wounds and administration of vaccine without administration of RIG; 12 cases with category II exposure had treatment of the wounds and administration of vaccine; and 7 cases with category III exposure received treatment of the wounds and administration of vaccine and RIG. It meant that 27.6% of the cases received inadequate PEP, and only 19 cases (6.0%) had a full regime of PEP.

Logistical regression analysis was performed to evaluate the factors that influenced PEP of rabies (Table 3). Dependent variables included local wound treatment, vaccination, and RIG administration. Independent variables included gender, age, occupation, species of attacking animal, animal vaccination status, category of exposure, and exposure site. Results of the analysis showed that the factors influencing wound treatment after rabies exposure included the category of exposure (odds ratio [OR] = 1.935) and the exposure site (OR = 0.557). The factors influencing vaccination administration included the category of exposure (OR = 3.437) and the exposure site (OR = 0.397). To clarify these results, we further analyzed the factors influencing rabies PEP (Table 4) and used multiple comparison to reveal the differences in rabies PEP with various categories of exposure and exposure sites. Results showed that the rate of wound treatment (*P *= 0.006) and vaccination (*P *< 0.001) was significantly higher in the cases with category III exposure than in those with category II exposure. The rate of wound handling (*P *< 0.001) and vaccination (*P *< 0.001) was markedly higher in the cases with head, face or neck exposure than in those with limb exposure. Since there were relatively few examples of trunk exposure, these were excluded from the analysis.

**Table 3:** Analysis of factors influencing use of human prophylaxis after rabies exposure

Y	X	B	**S.E**.	Wald	df	Sig. (*P*)	Exp(B)	95% C.I. for Exp(B)	
								
								Lower	Upper
Wound treatment	Category of exposure	0.660	0.222	8.822	1	0.003	1.935	1.252	2.991
	Exposure site	-0.584	0.132	19.451	1	0.000	0.557	0.430	0.723
	Constant	-0.197	1.112	0.031	1	0.860	0.821		

Vaccination	Category of exposure	1.235	0.327	14.232	1	0.000	3.437	1.810	6.528
	Exposure site	-0.925	0.169	29.879	1	0.000	0.397	0.285	0.553
	Constant	1.208	1.857	0.423	1	0.515	3.347		

**Table 4:** Analysis of factors influencing prophylaxis and treatment in the cases after exposure to rabies

				result	
				
Influential factors	Wound treatment rate (%)	Vaccination rate (%)	multiple comparison	Wound treatment	Vaccination
Exposure category					
I	14.3	12	I & II	*P *= 0.384	*P *= 0.925
II	20.7	11.4	I & III	*P *= 0.028	*P *= 0.044
III	32.2	31.7	II & III	*P *= 0.006	*P *= 0.000
Exposure site					
1. Head, face or neck	53.5	67.3	1 & 2	*P *= 0.000	*P *= 0.000
2. Upper limbs	27.9	16.5	1 & 3	*P *= 0.000	*P *= 0.000
3. Lower limbs	19.1	11.2	2 & 3	*P *= 0.021	*P *= 0.206

## Discussion

During the past 13 years, the burden of human rabies in China increased, especially in 2001 and 2003, with a small drop in 2005 and 2008. Starting in 2001, China stopped producing the concentrated rabies vaccine for human use, and replaced it with the purified rabies vaccine, including the primary hamster kidney cell or vero cell-culture rabies vaccines [8]. The price of the purified vaccine (156 RMB/person), was much higher than that of the previous concentrated vaccine (26 RMB/person). Some people exposed to rabies especially in rural areas were not vaccinated because they could not afford the expensive vaccine. This might be associated with the dramatic increase of human rabies incidence in 2001 [8,9]. Although the atypical pneumonia pandemic in 2003 in China caught the attention of the world, in the same period the number of deaths due to rabies far exceeded those caused by atypical pneumonia. The huge number of resources directed to atypical pneumonia, which perhaps influenced the control and prevention of rabies at that time. The reason for rabies incidence decreasing in 2005 may be due to rabies control being emphasized and strengthened by local governments in some high prevalence provinces, such as Hunan and Guangxi [10]. The rapidly and continuously increasing epidemic of rabies attracted the government of China, and a series of guidelines about rabies prevention were instituted and applied such as Technical Specifications of Rabies Control and Prevention in 2006 and Treatment Guidelines for Rabies Postexposure Prophylaxis of Humans in 2007, which maybe benefit the drop of rabies incidence in 2008.

Human rabies mainly distributed in the middle and southeast China and this distribution may be associated with the very high density of human populations and dog populations [11]. The range of rabies-affected areas has been extending in China, and spreading to the west and north. This may be due to the gradual development of the transportation network, increased transportation of rabid dogs with people among different areas, and related economic factors. For example, as the economic condition is improving in China, more and more people can afford to own a dog, and any areas that had no human rabies reports have reported outbreaks of rabies since 1996, such as ZJJ city, Hunan province, where 34 rabies cases were reported in 2005 while there were no case reports in 1996 to 2004.

The rabies cases predominately occurred in the rural areas of the counties, which perhaps related to the larger numbers, more free-ranging existence, and lower vaccination rate in dogs, lack of knowledge about rabies and poor economic conditions [2,12]. Affected populations were primarily farmers, children under 15 years old. The main seasons for rabies epidemics were summer and fall, when more frequent outdoor activities increased chance of human-rabid dog contact and exposure [13].

From the analysis of epidemiology characteristics of human rabies in recent years above, we know that China is now facing rabies outbreaks and the epidemic wave maybe relate to the change of rabies vaccine, application of related policies, and so on. To identify the more potential factors involved in the serious rabies emergence, we focused upon the three most seriously affected provinces, in which the two aspects of dogs and human PEP were analyzed in detail.

In China, dogs act as the main rabies virus infection source for human rabies [14]. However, dog rabies surveillance often hasn't been carried out because the dog isn't an important economic animal in china. Insufficient data was obtained to fully address the relationship between the dog rabies and human rabies. In southern China, there are custom of eating dog meat, which make it possible that many brain specimens could be collected at the same time that dogs are slaughtered in local restaurants. In these brain specimens from apparently healthy domestic dogs, rabies positive samples were identified by DFA. In fact, not only in China but also in other countries, the reports about identification or isolation of rabies virus in apparently healthy dogs repeated [14-19]. Although no date are available concerning how long such dogs survive and no adequate evidence are provided, the phenomenon may be explained that rabies virus has reached the central nervous system before clinical signs appear rather than there are carrier or asymptomatic rabies state exists. About the dogs purchased by restaurants, they will be slaughtered soon and no time to transmit rabies virus to other dogs and humans but the butchers. Up to now, the dog meat consumers are considered no risk of rabies infection for no related reports appear. In our research, the rabies infection rate in dogs throughout the three provinces (2.3%) was relatively lower than other surveys (3.9% to 17.9%) in China [18,19]. The discrepancies of infection rates is perhaps related to the sampling method (including the clinically suspicious rabid dogs or not), different sample sizes, detection methods, etc. Regardless of the infection rate, the data indicated that the disease in dogs prevailed in these regions, which represented an important cause of the high incidence of rabies in humans. But, there were no liner relationship between the rate of rabies infection in dogs and the incidence of human rabies, which illustrated that it was not the only factor influencing human rabies prevalence.

WHO has determined that vaccination coverage more than 70% is needed to sufficiently control canine rabies [5]. In 2006, the estimated number of dogs in China was over 75,000,000. According to the statistical data of the local veterinary department, the vaccination rate of dogs in most areas investigated in this study was much lower than 70%, which is not favourable to rabies control. As suggested in previous studies, low dog vaccination rates is one of the major contributing factors for the rabies epidemic in the three provinces [13,20,21]. The vaccination coverage in dogs needs tremendous improvement.

Except the status of dogs influencing the epidemic of human rabies, PEP is another factor. We explored the category of rabies exposure in 711 cases in the three provinces. Of the cases investigated, 91.0% were attacked by dogs and 6.5% were attacked by cats. Most of these dogs haven't been vaccinated, and vaccination of cats is paid even less attention than that of dogs. In China, there are no official administrative rules concerning the animals attacking humans. In some areas, the dog would be killed after attacking humans, with few receiving observation and rabies detection. The status of animals after attacking humans was not always obtained.

According to WHO, as long as victims bitten by animals receive proper PEP, rabies can be prevented completely [22]. In the 711 cases who died of rabies, 6.3% were classified category I contacts which should no risk of rabies. The phenomenon exposed a serious problem that some health care staff are not professional in rabies PEP. At the same time, the public ignorance of rabies should be notable, because 66.3% of victims did not seek medical services at all. 27.6% of the cases received inadequate PEP, which may contribute to the knowledge lack of rabies PEP of both medical staff and patients themselves. 6.0% of victims who had a full regime of PEP still died of rabies, may explained by the reduced quality of the vaccine due improper storage by the patients themselves after the first shot, as Han Si et al. analyzed [4]. The investigation showed that the rate of wound treatment in the cases with category III exposure was higher than in those with category II exposure, and the rates of wound treatment and vaccination administration in those cases with head, face or neck exposures were all higher than in those with only limb exposures. This illustrated that with serious exposure at sites close to the head, the patients were more likely to seek medical service, and that lighter exposure more often was ignored. All these reflected that many people were not aware of the risk of rabies and always had aleatory ideas. Thus, publicity and education on risk and prevention of rabies is necessary and important to control epidemic, and should be strengthen in endemic areas, especially in rural areas.

Another reason for low rate of rabies PEP might be related to poor economic conditions. In China, human rabies mainly occurred in rural areas with slower economic development. In the rural areas of three high prevalence provinces in our study, the average annual net income per person was 2,496 RMB in 2005, while the total of the cost for PEP/person is about 1150 RMB (the lowest retail price of the rabies vaccine is 150 RMB/person, and the average cost for RIG is 1,000 RMB/person), accounting for 46.1% of the annual income. Patients exposed to rabies in China were estimated to be between 1% and 10% of the population, and several million persons are expected to require PEP each year at a minimum [23]. To maximize efficiency of the limited subsidy and resource of human vaccines and rabies immunoglobulin for patients at real risk, PEP should be decided to initiate or withhold according to postmortem diagnosis of the biting animal. Therefore, establishing a systemic rabies diagnosis network is imperative. China government has aware of this problem, and some programs about rabies surveillance in animals especially in dogs have been carried out. It can be expected that reliable surveillance of dogs and aimed PEP service based on the diagnosis of the biting animal will effectively decreases the burden of human rabies epidemic.

In summary, only both animal rabies surveillance and control and human PEP are emphasized and strengthened at the same time, the serious human rabies epidemic in China at present could be effectively control, and the elimination of human rabies in China in the end will be possible.

## Conclusions

In recent years, rabies is reemerging and becoming a major public-health problem in China. The analysis of status of dogs and PEP in the Guangxi, Hunan and Guizhou province showed that unsuccessful control of dog rabies and inadequate PEP of humans were the main factors leading to the high incidence of human rabies in China. According to the study, there are following suggestions: (1) Strict control of free-ranging dogs and mandatory rabies vaccination should be enforced. (2)Establishing national animal rabies surveillance network is imperative. (3) PEP should be decided to initiate or withhold according to postmortem diagnosis of the biting animal. (4) The cost of PEP should be decreased or free, especially in rural areas. (5)Education of the public and health care staff should be enhanced.

## Competing interests

The authors declare that they have no competing interests.

## Authors' contributions

MS and QT conceived of and designed this study, and drafted the manuscript. XYT and HL collected the data, and performed the statistical analysis. ZJM, DMW, SHG, CER, ZJF and GDL made significant contributions to this work by providing assistance and helped in the data collection, data manipulation and analysis. All authors read and approved the final manuscript.

## Pre-publication history

The pre-publication history for this paper can be accessed here:

http://www.biomedcentral.com/1471-2334/9/210/prepub
